# Construction of a Gaussian Process Regression Model of Formamide for Use in Molecular Simulations

**DOI:** 10.1021/acs.jpca.2c06566

**Published:** 2023-02-09

**Authors:** Matthew L. Brown, Jonathan M. Skelton, Paul L. A. Popelier

**Affiliations:** Department of Chemistry, The University of Manchester, Oxford Road, Manchester M13 9PL, Britain

## Abstract

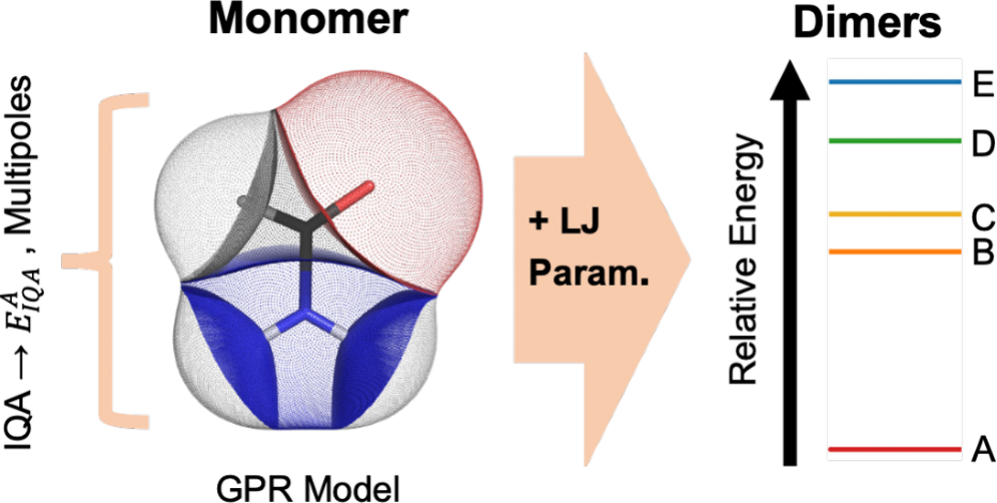

FFLUX, a novel force field based on quantum chemical topology, can perform molecular dynamics simulations with flexible multipole moments that change with geometry. This is enabled by Gaussian process regression machine learning models, which accurately predict atomic energies and multipole moments up to the hexadecapole. We have constructed a model of the formamide monomer at the B3LYP/aug-cc-pVTZ level of theory capable of sub-kJ mol^–1^ accuracy, with the maximum prediction error for the molecule being 0.8 kJ mol^–1^. This model was used in FFLUX simulations along with Lennard-Jones parameters to successfully optimize the geometry of formamide dimers with errors smaller than 0.1 Å compared to those obtained with D3-corrected B3LYP/aug-cc-pVTZ. Comparisons were also made to a force field constructed with static multipole moments and Lennard-Jones parameters. FFLUX recovers the expected energy ranking of dimers compared to the literature, and changes in C=O and C–N bond lengths associated with hydrogen bonding were found to be consistent with density functional theory.

## 1 Introduction

Classical force fields have been used for many years to investigate chemical problems in a wide variety of research areas. They are an ideal tool due to their low computational cost, which allows large systems to be studied on timescales that *ab initio* methods cannot access. Despite their widespread application, classical force fields have several limitations. One is the use of point charges, which still appear in the most popular methods despite evidence that multipolar electrostatics improves accuracy (see ref (1) for a review). A shift to the use of point or extended multipole moments has been seen in a variety of models such as AMOEBA+,^2,3^ SIBFA21,^4^ DIFF,^5^ MASTIFF,^6^ Slater-FF,^7^ and GEM.^8^ Simulation codes such as DMACRYS,^9^ DL_MULTI,^10^ OpenMM,^11^ and Tinker-HP^12^ all allow for use of multipoles, with OpenMM and Tinker-HP being in active development. DMACRYS has been used for the optimization of crystal structures for many years, employing a distributed multipole representation of the charge density of rigid molecules with calculations potentially up to the hexadecapole moment. Another limitation of classical force fields is the parameterization of bond, angular, and torsional terms, which differ between force fields and have varying levels of accuracy. These approximations, among others, mean that classical force fields are not accurate enough in practice to determine the typically few kJ mol^–1^ energy differences between polymorphs of crystal structures.^13^

FFLUX^14,15^ is a next-generation force field that aims to address these shortcomings. FFLUX is based on the quantum theory of atoms in molecules (QTAIM)^16^ and the interacting quantum atoms (IQA)^17^ energy partitioning scheme. Both QTAIM and IQA fall under the umbrella of quantum chemical topology (QCT), a collection of approaches that share the idea of a gradient vector field partitioning a quantum mechanical function. FFLUX utilizes Gaussian process regression (GPR)^18^ machine learning models trained on QCT data. These models can quickly predict atomic energies and multipole moments (up to the hexadecapole) in real time during molecular dynamics (MD) simulations using just the coordinates describing an atomic environment. GPR models offset the computational cost of QCT calculations and allow for fully flexible molecules with polarizable atomic multipole moments responsive to the geometry of a system. The models have been shown to be transferable^19^ with near *ab initio* levels of accuracy.^20,21^ Conceptually, the GPR models have a “plug and play” nature, allowing FFLUX to be applied to a broad range of systems, which turns out to be a challenge for other state-of-the-art force fields such as MB-pol.^22^

Hydrogen bonds play an important role in the structure, dynamics, and properties of many systems, and it is therefore important for computational methods to predict them accurately. For example, the dependence of the properties of molecular crystals on the arrangement of molecules in the structure is often due to the different patterns of intra- and intermolecular hydrogen bonding they facilitate. This is among the factors that lead to polymorphism, whereby molecules can adopt multiple crystal forms with different properties. For example, the conformational polymorphs of the HIV drug ritonavir contain different networks of hydrogen bonds that play a part in the different solubilities of the two forms.^23^ Hydrogen bonding has previously been studied with QTAIM to understand the covalent interactions involved^24^ and to establish the origins of resonance-assisted hydrogen bonding.^25^

The amide functional group appears regularly in biological and pharmaceutical molecules where it gives rise to a variety of hydrogen-bond geometries in clusters and in the solid state. The range of geometries available offer a suitable test of the ability of FFLUX to predict structural properties of systems larger than the monomeric GPR models are trained for. The test system that we use to validate the FFLUX methodology is formamide, which is the smallest amide with just six atoms. This choice is justified because of its well-studied potential energy surface (PES) and its small size making the construction of a good quality GPR model feasible in a shorter time frame than for larger amides. We note that FFLUX is capable of simulating flexible systems.

**Figure 1:**
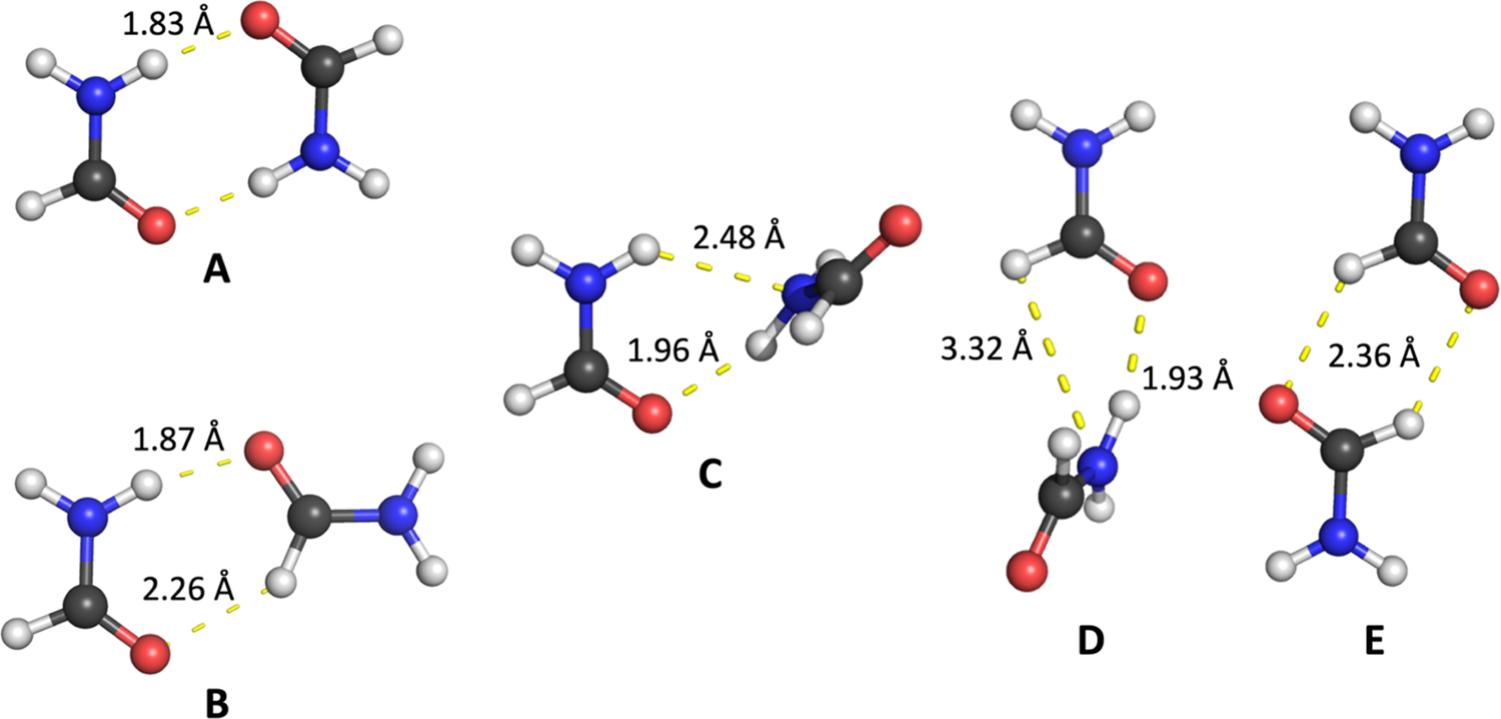
Geometries of the formamide dimers, identified previously,^29^ which were optimized at the B3LYP-D3/aug-cc-pVTZ level of theory. Hydrogen bond lengths are labeled.

The structure of formamide itself has also been studied as recently as 2016 with Møller–Plesset second-order perturbation theory (MP2) and CCSD(T) calculations.^26^ Five minima (*A*–*E*) on the PES of the formamide dimer have been identified using MP2^27−30^ and studied in matrix isolation experiments.^30^Figure 1 shows the structures of these minima, optimized at the B3LYP/aug-cc-pVTZ level of theory with the D3 correction.^31^ Coordinates for the monomer and dimers *A*–*E* are given in Section 1 in the Supporting Information (SI) in Tables S1.1–S1.6 along with ball and stick images in Figures S1.1–S1.6. The hydrogen bonding in formamide dimers has also been studied extensively beyond the aforementioned references,^24,32−34^ with formamide dimers used as a test system for an additional term in the MM3 force field to improve the directionality of the hydrogen bonds.^32^ In principle, FFLUX does not require any such modifications because hydrogen bonds are naturally captured by the electrostatic multipole moments predicted by the GPR models.

Here, a formamide GPR model is constructed and used in conjunction with a Lennard-Jones model for dispersion and repulsion in FFLUX MD simulations of dimers for the first time. The relative energy ordering and hydrogen bonding geometries of the dimers are compared to B3LYP-D3/aug-cc-pVTZ calculations, where the D3 correction allows for fairer comparison to FFLUX optimizations with parameterized dispersion. A rigid-body force field based on static IQA moments is constructed with the same nonbonded parameters and used to test the effect of the flexible multipole moments in FFLUX. Finally, the variation in the C=O and N–H bond lengths due to the formation of hydrogen bonds in the simulations is tested.

## 2 Methods

The FFLUX force field is implemented in DL_FFLUX, which is derived from DL_POLY 4.^35^ The force field itself replaces the electrostatic and traditional bonded terms in a force field with atomic energies and multipole moments predicted by the GPR model. For simulations of systems larger than a monomer, nonbonded parameters are used as a measure of dispersion and repulsion although, in theory, these can also be trained for in the GPR model. The methodology behind FFLUX has been explained elsewhere,^14,15^ but a brief overview is provided as part of this work.

### 2.1 QTAIM

**Figure 2:**
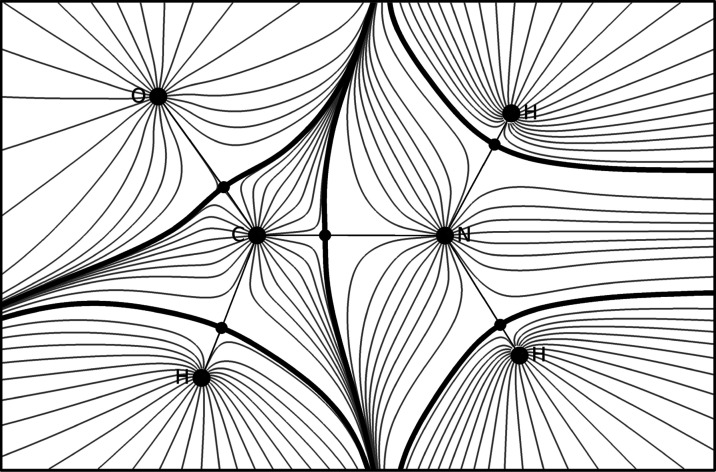
Gradient paths (gray lines) for a formamide monomer. Large circles represent maxima in the electron density (nuclei). Interatomic surfaces are represented by thick black lines, while the small disks that lie on these surfaces are saddle points named bond critical points.

At the heart of FFLUX is the topological atom, which emerges naturally as a subspace of the electron density when a gradient vector field is applied. Each topological atom contains a portion of the total electron density and an attractor (the nucleus), which attracts trajectories of the electron density gradient termed gradient paths. The boundaries of the topological atoms are defined by interatomic surfaces or zero-flux surfaces obeying eq 1
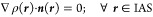
1These surfaces are made up of gradient paths for which a normal vector to the surface ***n***(***r***) is orthogonal to a point ***r*** belonging to the interatomic surface (IAS) and which are not crossed by any other gradient paths. Figure 2 shows the gradient paths for a formamide monomer and highlights various topological objects.

Topological atoms are obtained without the use of a reference density and are also space-filling and nonoverlapping by construction. This allows for the recovery of the wavefunction energy without corrections for penetration energies.

### 2.2 Interacting Quantum Atoms (IQA)

The IQA method is an energy decomposition scheme that extends the reference-free, virial-based QTAIM partitioning scheme by now allowing nonequilibrium geometries to be partitioned into atomic energies. The IQA energy decomposition is achieved by partitioning one- and two-electron density matrices into atomic energies that sum to the total wavefunction energy.

Atomic IQA energies, *E*_IQA_^*A*^, can be broken down into the intra- and interatomic contributions given in eq 2
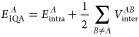
2where *E*_intra_^*A*^ and *V*_inter_^*AB*^ are the intra- and interatomic energies, respectively. These two terms can be broken down further according to
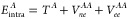
3
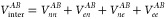
4The subscripts indicate either a nuclear (*n*) or electronic (*e*) interaction of the corresponding superscript atoms *A* or *B*. The intra-atomic energy in eq 3 is the sum of the kinetic energy of the electrons (*T*^*A*^) and nuclear–electron (*V*_*ne*_^*AA*^) and electron–electron (*V*_*ee*_^*AA*^) potential energies. The interatomic terms in eq 4 comprise the nuclear–nuclear and electron–electron interactions between pairs of topological atoms (*V*_*nn*_^*AB*^ and *V*_*ee*_^*AB*^) as well as the potential energies from the nucleus of one atom interacting with the electrons of the other and *vice versa*. *V*_*ee*_^*AB*^ can be further partitioned into Coulomb and exchange-correlation energies *V*_coul_ + *V*_xc_. The classical terms (that is, purely electrostatic, excluding pure quantum effects) can then be grouped together as *V*_cl_ to write *V*_inter_^*AB*^ as
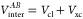
5

### 2.3 Gaussian Process Regression in FFLUX

GPR models are used in FFLUX to capture short-range interactions, with each atom in a molecule having its own, separate models that predict its atomic energies and atomic multipole moments. Each model has “knowledge” of its surroundings such that the predictions made by a model *M*_*A*_ depend not only on atom *A* but also on the other atoms in the system being modeled. In this context, the “system” is the formamide monomer, but in general, it is the molecule or assembly of molecules that the GPR model is being trained for. The training is completed using the in-house automated pipeline ICHOR,^36^ written in Python, which makes use of adaptive sampling^21^ (a.k.a. active learning).

ICHOR completes the following series of steps to create the GPR models required for FFLUX simulations:

(1) Generate a series of molecular configurations.
(2) Calculate the wavefunction of each configuration using GAUSSIAN09.^37^
(3) Calculate *E*_IQA_^*A*^ and multipole moments for each atom in each configuration using AIMAll.^38^
(4) Map these properties to geometric features by Gaussian process regression, which is implemented in the in-house code FEREBUS.
(5) Use active learning to identify the best points to add to improve the model.

Models can be generated following “per-system” and “per-atom” approaches where the same training set is used for each of the atoms in the system (“per-system”) or where each atom has its own unique training set (“per-atom”).^39^

Molecular configurations can be generated in a variety of ways. In the past, normal mode distortions^40^ and *ab initio* molecular dynamics (AIMD)^21^ simulations have been used to generate points, but in this work, the sample pool was generated from a classical MD (AMBER) simulation of formamide at 300 K with one million steps. This provided a sample pool that was orders of magnitude larger than could feasibly be obtained with AIMD in a similar time frame.

The structures generated were split into three sets: (i) a training set (initially 36 points), which is the set of points that the model is trained on; (ii) a validation set (500 points) used to test the model; and (iii) a sample set (100,000 points) from which the active learning finds points to add to the model for improvement, upon each iteration of training. The initial training set was generated by selecting points from the trajectory representing the minimum, maximum, and mean of each feature. The points in the sample and validation sets were randomly selected from the remaining geometries in the trajectory. Wavefunctions of each geometry were then calculated at the B3LYP/aug-cc-pVTZ level of theory using GAUSSIAN09^37^ and IQA decompositions were performed by the program AIMAll.^38^

The GPR model training is implemented using the in-house program FEREBUS,^41^ which takes an input vector containing *N*_train_ training points. Each training point is an *N*_feat_-dimensional vector corresponding to an output (atomic IQA energy or atomic multipole moment) collected in a vector ***y***. The *N*_feat_ input features are defined in a set of atomic local frames (ALFs)^42^ with the origin being the atom for which the model is trained (atom *A*). Two atoms are required to fix the *x*-axis (atom *A*_*x*_) and the *xy*-plane (atom *A*_*xy*_), which are determined by the Cahn–Ingold–Prelog rules. The *x*-axis and *xy*-plane are defined by the atom with highest and second highest priority, respectively. The *z*-axis is then constructed orthogonally to form a right-handed axis system. The first three features are: (i) the distance between the ALF’s origin and *A*_*x*_, (ii) the distance between the ALF’s origin and *A*_*xy*_, and (iii) the *A*_*x*_–*A*–*A*_*xy*_ angle. All other atoms are then defined by spherical coordinates relative to the ALF.

For the GPR models used in FFLUX, the similarity between two points in the training set is measured using a modified radial basis function (RBF) kernel. This similarity is expressed in a covariance matrix that depends on the hyperparameter θ_*k*_, which has units that are the reciprocal of the corresponding feature to maintain the dimensionless exponential. Every third feature is a cyclic feature that ranges from −π to π and a correction *r*_*k*_(***x***_*k*_,***x***_k_^*^) is introduced to the RBF kernel to account for this. Hyperparameters are optimized by maximizing a concentrated log-likelihood, and subsequently used to recalculate the covariance matrix and make predictions as shown in eq 6
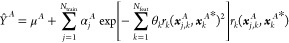
6
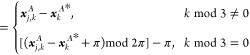
Here, *Ŷ*^*A*^ is the predicted energy or multipole moment of the topological atom *A*, μ^*A*^ is the average value of the output for all of the training points, and *a*_*j*_^*A*^ is the weight of the *j*th training point. The vectors ***x***_*j*_^*A*^ and ***x***^A*^ represent the *k* features of the *j*th training point and the unseen point, respectively. The formamide model constructed in this work used the per-system approach, with each atom having 1506 training points and 3*N* – 6 = 12 features.

### 2.4 Ewald Summation

The predicted atomic energy values (*Ê*_IQA_^*A*^) can be used to calculate intramolecular forces by taking the negative gradient with respect to displacement as given by eq 7
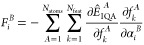
7where *F*_*i*_^*B*^ is the *i*th force component on atom *B*, α_*i*_^*B*^ is its global *i*th coordinate, and *f*_*k*_^*A*^ are the features of atom *A* exerting a force on atom *B*.^42^ This procedure replaces the use of harmonic bond and angle potentials seen in many traditional force fields allowing for a PES that lies closer to quantum mechanics.

While the short-range (intramolecular) electrostatic interactions are captured by *E*_IQA_^*A*^, predicted multipole moments are used to calculate long-range electrostatic interactions with the smooth particle mesh Ewald (SPME) method.^43^ SPME is commonplace in many force fields but had to be adapted for use with the flexible multipole moments present in FFLUX. Here, the term flexible means that the moments can change with the geometry of the system, and because of this, additional terms arise in the calculation of electrostatic forces. The derivation^59^ of these terms has been validated using two different limits of the SPME sum and comparisons to other methods.

## 3 Results and Discussion

### 3.1 Performance of the GPR Model

To determine the accuracy of the formamide GPR model at predicting the energies and multipole moments one can look at the prediction error (PE) for each atom, which is given by
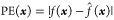
8In the case of the energies, *f*(***x***) is the IQA energy of an atom and *f̂*(***x***) is the energy predicted by the model. The prediction error of the system can then be calculated as the sum of the prediction errors over all of the atoms in the system or as the absolute difference between the total IQA energy of the system and the predicted energy of the system allowing for cancellation of errors.

**Figure 3:**
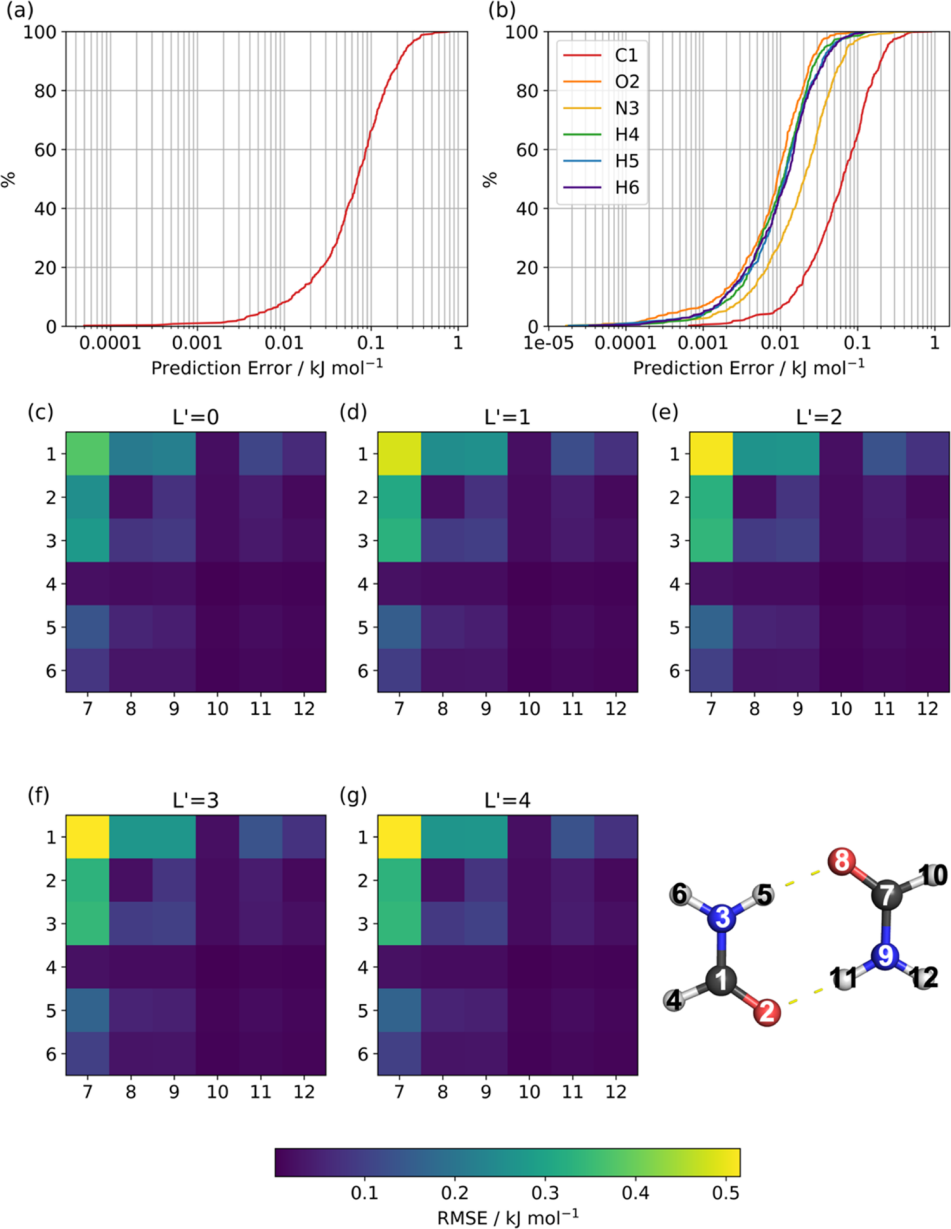
Accuracy of the 1506-point formamide GPR model. (a) *S*-curve showing the error in the predicted IQA total energies for each configuration in a 500-point training set. (b) *S*-curves showing the error in the predicted IQA energies for each atom. In both (a) and (b), the prediction errors are plotted against percentile. (c–g) Root-mean-squared error (RMSE) in the predicted multipole moments, converted to electrostatic energies, for intermolecular atom–atom interactions across a 500-point validation set of formamide dimers generated at 300 K. The indices on the axes of each heatmap refer to the atom indices depicted in this figure. Electrostatic energies were calculated using truncations of the multipole expansion denoted by the value *L*′, which indicates the highest rank present in the simulation. For example, an *L*′ value of 0 indicates monopoles (charges) only, whereas a value of 4 indicates interactions up to the hexadecapole moment are used.

Prediction errors that allowed for cancellation of errors were calculated for each of the 500 geometries in the validation set. The errors are arranged in ascending order and plotted against percentile to produce *S*-curves, so-called due to their sigmoidal shape, as shown in Figure 3a,b. The *S*-curves show a cumulative error distribution that intersects the 50% percentile at 0.07 kJ mol^–1^ indicating that the formamide model predicts the energies of half the validation geometries with an error less than 0.07 kJ mol^–1^. The maximum error, where the curve hits 100%, is 0.8 kJ mol^–1^ and is comfortably below the nominal 1 kcal mol^–1^ (4.2 kJ mol^–1^) threshold of chemical accuracy. Using the sum of absolute atomic errors, the maximum error of the model was still below this threshold with a value of 1.1 kJ mol^–1^ (50% of errors below 0.14 kJ mol^–1^).

The accuracy of the predicted multipole moments can also be analyzed using *S*-curves, examples of which are given in Section 2 in the SI for charges (Figure S2.1) and dipoles (Figure S2.2). In particular, Figure S2.1 shows that the worst predicted atom, which is carbon, shows a maximum error of 1.5 milli-electrons (me), while 99% of predictions have an error of less than 1 me. While this information is important if one wants to study charge transfer effects within a formamide monomer, the errors are best converted to electrostatic energies to assign the charge errors an interpretation in terms of intermolecular electrostatic energies. If the latter is confined to charge–charge (or monopole–monopole) energy only, then eq 9 is a convenient means^19^ to convert the error in charge *A*, denoted Δ*Q*_00_^*A*^, into the energy, Δ*E*_*AB*_, of *A* interacting with a charge *B* over a distance *R*_*AB*_
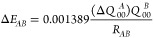
9where the prefactor has been adjusted for the charges to be expressed in milli-electrons, the distance in Å, and the energy in kJ mol^–1^. A charge error of 1 me, in the presence of a probing charge of a generous 1500 me at a very short distance of 2 Å, yields an energy error of 1.0 kJ mol^–1^.

Such error analysis can be made more sophisticated by converting all multipole moments (not just the charges) to electrostatic energies between two formamide molecules. A validation set of 500 dimers was taken from a CP2K simulation of the formamide dimer at 300 K that we had available from other work. The electrostatic energies were calculated for each intermolecular atom–atom interaction using the predicted multipole moments. The predicted electrostatic energies can then be compared to the energies calculated using the exact multipole moments for each monomer in the dimer obtained from the program AIMAll, allowing the error for each interaction across the validation set to be calculated. Figure 3c–g shows these errors as heatmaps for each given value of *L*′, where *L*′ is the highest-rank multipole moment present in the simulation. We find that the electrostatic energies are predicted well with the RMSEs for all of the atom–atom interactions below 1 kJ mol^–1^. In both the electrostatic and the IQA energies, the carbon atom contributes most to the error. This is likely due to it being bonded to two nonhydrogen atoms that can cause larger distortions to the carbon topological atom, making it more difficult to predict.

FFLUX requires no approximations to bond, angle, or torsional energies but instead relies on the GPR model to predict the intramolecular PES. To test the performance of the energy GPR models in finding the minimum-energy monomer, a geometry optimization can be performed using FFLUX and compared to the training level of theory (*i.e.*, B3LYP/aug-cc-pVTZ). The energy of the optimized formamide monomer predicted by GAUSSIAN using B3LYP/aug-cc-pVTZ is −446265.0 kJ mol^–1^, while FFLUX predicts an energy of −446264.9 kJ mol^–1^. The formamide model thus achieves sub-kJ mol^–1^ accuracy with an error of only 0.1 kJ mol^–1^ (2 × 10^–5^%). Geometrically, the maximum difference in bond length between the two methods is 6.2 × 10^–4^ Å while the largest change in bond angles is 0.06 degrees. Tables S3.1 and S3.2 of Section 3 in the SI give the differences in all of the bond lengths and angles in the monomer. Such small differences show that the GPR model is allowing FFLUX to predict the minimum energy structure well.

A test for the charge and dipole models is how well the molecular dipole moment is predicted. The dipole moment calculated for the optimized gas-phase monomer in GAUSSIAN09 is 3.943 D at the B3LYP/aug-cc-pVTZ level of theory. The dipole moment predicted by FFLUX differs by less than 0.1% with a value of 3.941 D, which confirms that the atomic charge and atomic dipole GPR models are capable of reproducing the molecular dipole moment at the training level of theory, as seen in previous work.^44^

### 3.2 FFLUX-Optimized Dimers

#### 3.2.1 Computational Details

Two sets of dimer optimization were performed using FFLUX. In the first set, *ab initio* optimized dimers were used as starting geometries. These were obtained by taking structures approximating the five minima identified previously^29^ and optimizing them at the B3LYP-D3/aug-cc-pVTZ level of theory (shown in Figure 1). In the second set, random starting geometries were used. These optimizations allowed us to check that no unexpected minima are found using the chosen nonbonded potential and to probe the intermolecular PES facilitated by the chosen Lennard-Jones parameters. Optimizations were also performed using a rigid-body force field constructed using permanent multipole moments from an IQA analysis of the B3LYP/aug-cc-pvTZ optimized monomer and the same Lennard-Jones parameters (IQA + LJ). This allowed comparison to a force field with a similar form to other multipolar force fields that utilize rigid bodies. The multipole moments for each atom are given in Section 4 in the SI.

Initial dimer geometries were placed into a 50 Å × 50 Å × 50 Å cubic box. As DL_FFLUX is built onto DL_POLY it can make use of several of the routines available in the DL_POLY code. For the optimization of formamide dimers, the DL_POLY 0 K (zero Kelvin) optimizer was used, specified by the “zero” directive. This is equivalent to an MD simulation at low temperature where the molecules move in the direction of the computed forces but are not allowed to gain a velocity larger than they would at 10 K. Simulations were run for 3000 timesteps (each step being 1 fs) and the optimized structure was taken as the final timestep. For the optimizations starting from random geometries, a more generous 15,000 timesteps was given to allow the simulations to converge. A Hoover thermostat with a relaxation constant of 0.02 ps was used and optimizations were run at *L*′ = 3. As noted previously *L*′ is the highest-rank multipole moment present in the simulation, so *L*′ = 3 includes monopole (*l* = 0), dipole (*l* = 1), quadrupole (*l* = 2), and octupole moments (*l* = 3) together with all possible interactions between them (in a square matrix rather than the familiar triangular multipole-multipole combinations controlled by *L* = *l*_*A*_ + *l*_*B*_ + 1). The same electrostatic rank and settings were used for the IQA + LJ optimizations.

Convergence of the optimizations was determined by two criteria. The first criterion was that the gradient between the final, *N*th, step and the *N* – 1000th step had to be less than (or equal to) 1 × 10^–4^ kJ mol^–1^ timestep^–1^ such that the energy change over the last 1000 steps was less than 0.1 kJ mol^–1^. The second criterion was that the deviation in energy from the straight line connecting the *N*th and the *N* – 1000th step had to be less than 0.1 kJ mol^–1^ such that there was no significant change in energy from the converged value between the two points. This deviation was calculated by taking the sum of square differences between the energy at each of the 1000 points and the energy at the equivalent point on the line connecting the *N*th and the *N* – 1000th step. This was then divided by 1000 (the number of steps) and the square root was taken, effectively calculating an RMSE. Both criteria had to be met for convergence to be achieved.

Going from monomeric calculations to dimeric (and larger system size) calculations requires additional nonbonded parameters to be used, providing a measure of dispersion and repulsion. These parameters are required because the monomeric GPR model only “knows” about itself, which means upon meeting another molecule in a simulation the model can only predict how to interact electrostatically with no way to predict the repulsion or dispersion. Short-range polarization is captured by the GPR model as multipole moments can change with geometry, but there is no explicit long-range (intermolecular) polarization in the model. The latter arises when the electron density is allowed to change (caused by the presence of other molecules) even when the geometry of the molecule remains fixed. This is a weakness of monomeric modeling and can be accounted for using *N*-meric model, where *N* is the number of molecules in the model.

To account for the lack of repulsion and dispersion nonbonded parameters from the FIT,^9,45^ GAFF,^46^ Hagler,^47,48^ OPLS/AA,^49,50^ and W99^51^ force fields were tested to identify a starting point before adapting the parameters for the FFLUX force field. Ultimately, the Hagler parameters were chosen as a starting point because these found all of the dimers apart from dimer *E*. Simulations using the OPLS/AA parameters obtained dimers *B*, *D*, and *E* but failed to obtain the global minimum (dimer *A*) or dimer *C*. This was due to the parameters not working well with the multipolar models and causing overbinding of the dimers and optimizations to crash. The RMSEs of the dimers obtained with the OPLS/AA parameters were generally larger than the RMSEs of dimers optimized using the original Hagler sets: dimer *B* was obtained with an RMSE of 0.12 Å compared to 0.05 Å using the Hagler set, while dimer *D* was obtained with an RMSE of 0.25 *versus* 0.18 Å. The FIT, GAFF, and W99 parameter suffered from a similar problem leading to crashes, which meant none of the five minima were found. This made the Hagler parameter set the best starting point. These parameters were optimized for use in *L*′ = 3 simulations by perturbing the initial values and comparing hydrogen bond lengths and angles in the FFLUX-optimized dimers to the B3LYP-D3/aug-cc-pVTZ dimer geometries as well as looking at RMSE. More details are given in Section 5 in the SI, including a list of the parameters used in the FFLUX simulations in Table S5.1. A cutoff of 12 Å was used for the electrostatics and Lennard-Jones interactions.

Energies of the dimers from FFLUX and IQA + LJ were collected to compare the relative energy rankings to B3LYP-D3/aug-cc-pVTZ, and RMSEs were calculated after applying the Kabsch algorithm to find the optimal mapping of the dimers onto one another. The parameters for the SPME sum were fixed by setting the DL_POLY keyword “spme sum α *k*1 *k*2 *k*3” to “spme sum 0.000001 20 20 20” where α is the Ewald convergence parameter (in Å^–1^) and the three *k* values specify the dimensions of the SPME charge array (effectively defining the range of the reciprocal space sum).

#### 3.2.2 FFLUX-Optimized Dimers Starting from Minima

The literature^27−30^ consistently reports that the relative energy ranking of dimers *A* to *E* obtained using MP2 is *A* < *B* < *C* < *D* < *E*. The calculations performed in this work using B3LYP-D3/aug-cc-pVTZ agree with this ranking.

**Figure 4:**
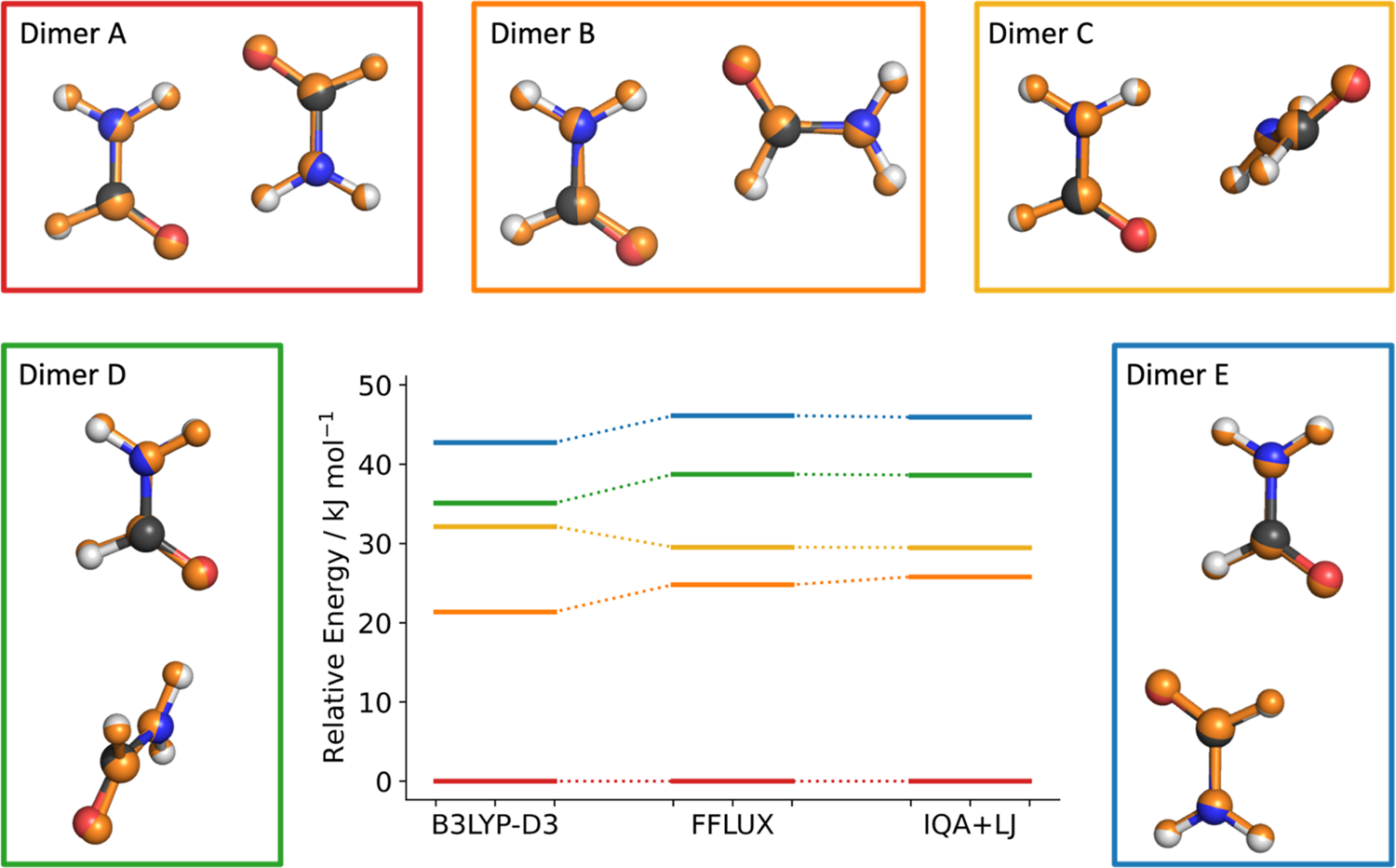
Structures and relative energies of the five formamide dimers *A*–*E* obtained with B3LYP-D3/aug-cc-pVTZ, FFLUX, and IQA + LJ. The B3LYP-D3-optimized geometries (atom colors) of each dimer are shown overlapped with the FFLUX-optimized geometry (orange) in colored boxes corresponding to the colors of the lines on the relative energy plot: *A*—red; *B*—orange; *C*—yellow; *D*—green; and *E*—blue.

FFLUX was used to geometry-optimize the dimers using the B3LYP-D3/aug-cc-pVTZ geometries as initial geometries. Note that both the intramolecular and intermolecular coordinates change in this optimization process as FFLUX allows for full molecular flexibility. The hydrogen bond lengths predicted by FFLUX are shown in Table 1 together with the RMSE between the B3LYP-D3/aug-cc-pVTZ and FFLUX geometries, and hydrogen bond angles are given in Table 2. The energies of the optimized geometries (taken as the final point in the zero Kelvin MD) were then used to obtain the relative energy ranking and compared to the DFT ranking. This comparison is shown in Figure 4, and relative energies are given in Section 6 in the SI (Table S6.1).

**Table 1:** Hydrogen Bond Lengths in Formamide Dimers Optimized Using B3LYP-D3/aug-cc-pVTZ, FFLUX, and IQA + LJ^a^

		hydrogen-bond lengths (Å)				
dimer	method	O···HN	O···HC	N···HN	N···HC	RMSE (Å)
*A*	B3LYP-D3	1.83				
	FFLUX	1.83				0.03
	IQA + LJ	1.83				0.02
*B*	B3LYP-D3	1.87	2.26			
	FFLUX	1.94	2.11			0.07
	IQA + LJ	1.94	2.10			0.09
*C*	B3LYP-D3	1.96		2.48		
	FFLUX	1.92		2.44		0.03
	IQA + LJ	1.93		2.44		0.02
*D*	B3LYP-D3	1.93			3.32	
	FFLUX	1.89			3.36	0.08
	IQA + LJ	1.90			3.36	0.06
*E*	B3LYP-D3		2.36			
	FFLUX		2.31			0.02
	IQA + LJ		2.31			0.02

a The RMSEs between the force-field-optimized and B3LYP-D3-optimized geometries are also given.

**Table 2:** Hydrogen Bond Angles in Formamide Dimers Optimized Using B3LYP-D3/aug-cc-pVTZ, FFLUX, and IQA + LJ

		hydrogen-bond angles (°)				
dimer	method	CO···H	NH···O	CH···O	NH···N	CH···N
*A*	B3LYP-D3	120.9	173.2			
	FFLUX	118.3	175.4			
	IQA + LJ	118.5	175.6			
*B*	B3LYP-D3	108.1/114.8	167.3	142.5		
	FFLUX	105.3/116.1	164.4	147.5		
	IQA + LJ	105.3/115.6	163.9	148.0		
*C*	B3LYP-D3	112.7	158.0		134.1	
	FFLUX	111.8	160.0		134.0	
	IQA + LJ	111.8	159.3		133.8	
*D*	B3LYP-D3	113.6	166.2			91.3
	FFLUX	114.0	169.9			89.3
	IQA + LJ	114.2	169.7			89.6
*E*	B3LYP-D3	99.1		138.6		
	FFLUX	99.6		138.3		
	IQA + LJ	99.6		138.1		

As shown in Figure 4, the structural differences between FFLUX- and DFT-optimized geometries are small with all geometries having RMSEs ≤ 0.08 Å. Hydrogen bond lengths are generally predicted well with all but one difference below 0.1 Å, with the largest difference being 0.15 Å in dimer *B*. This hydrogen bond is shortened in the FFLUX-optimized geometry and, combined with generally larger errors in the hydrogen bond angles, results in a larger RMSE. Ultimately the hydrogen bonding comes about naturally in FFLUX simulations due to the electrostatics, which are counterbalanced by the chosen nonbonded parameters. The differences here can be put down to the Lennard-Jones parameters having been adapted to obtain a good coverage of all five dimers rather than having an individual set of parameters for each dimer. The Lennard-Jones parameters act as an approximation to the true dispersion and repulsion that would exist in the dimer as the formamide GPR model is only aware of the monomer and how its atoms interact with each other, and not how molecules would interact other than through multipolar electrostatics.

The energy ranking predicted by FFLUX recovers the accepted ranking in the literature, expanding the energy difference between dimers *C* and *D* while diminishing the energy difference between *B* and *C*. Nevertheless the relative energies are obtained within 1 kcal mol^–1^ (4.2 kJ mol^–1^) of the B3LYP-D3/aug-cc-pVTZ relative energies, often quoted as chemical accuracy. Given that the monomeric energy is predicted almost exactly, the energy differences in the dimer simulations must arise from the terms that are not present in the monomeric simulations.

Comparing the FFLUX results to the IQA + LJ results, there is very little difference between the two methods with both obtaining B3LYP-D3/aug-cc-pVTZ geometries with similar accuracy. We expect that the small difference between the force field methods is due to the rigidity of formamide. This means that multipole moments will not change significantly in simulations such that similar results are obtainable with a rigid body multipolar force field. When applied to a more flexible system where multipole moments would potentially change more, we believe FFLUX would show a more significant improvement in accuracy from the rigid body model. This will be tested in future work.

#### 3.2.3 FFLUX Simulations Starting Away from Minima

For a more thorough test of the intermolecular PES as predicted by FFLUX with the chosen nonbonded parameters, 100 optimizations were performed. Each optimization started from an initial geometry away from the five known minima, with geometries generated by placing two *ab initio* optimized gas-phase monomers centered at random points, between 2 and 5 Å apart, and rotating them by a random angle about either the *x*, *y* or *z* axes. Energy minimizations were carried out as described in Section 3.2.1 for the FFLUX and IQA + LJ force fields. B3LYP-D3/aug-cc-pVTZ optimizations of the random geometries were performed in GAUSSIAN, at first using a steepest decent optimization, before using the default Newton–Raphson optimization.

**Figure 5:**
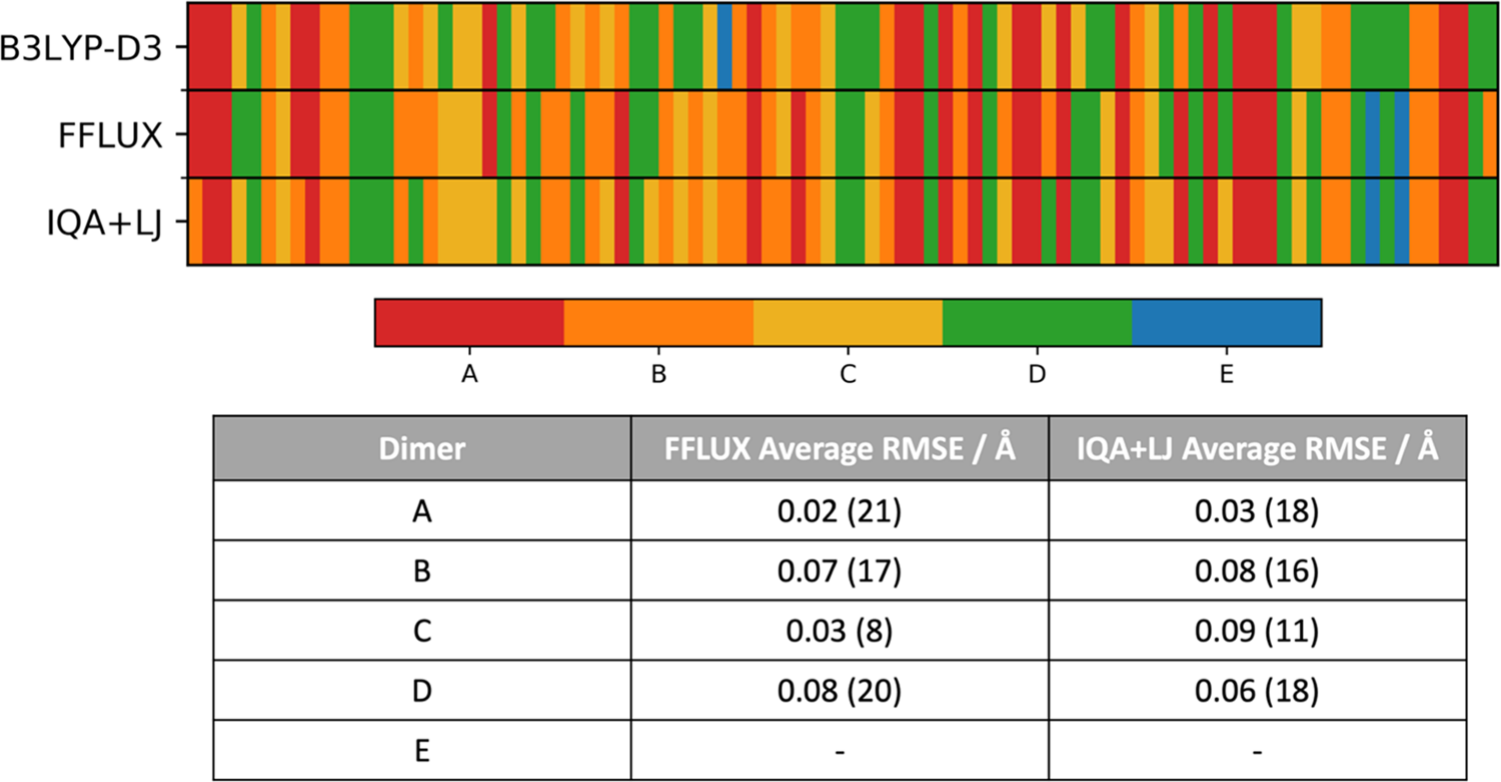
Summary of the 89 geometry optimizations starting from random geometries where each segment shows a color corresponding to the optimized geometry: *A*—red, *B*—orange, *C*—yellow, *D*—green, and *E*—blue. FFLUX predicts the correct geometry in 74% of the optimizations compared to B3LYP-D3 while IQA + LJ predicts 71% correctly. Average RMSEs of the optimized structures that matched the B3LYP-D3 optimizations are given with the number of correct matches shown in parentheses. Dimer E had no correct matches so no values are given.

Out of the 100 optimizations, 8 did not converge in FFLUX calculations within the given number of timesteps and 3 geometries were optimized to transition states in the B3LYP-D3 optimizations (confirmed by frequency calculations). These were excluded from the final analysis leaving 89 geometries for comparison. Each dimer was identified by calculation of the RMSE with respect to the B3LYP-D3 geometries. If the RMSE was below a generous threshold of 0.2 Å, the optimized dimer was identified as the corresponding geometry for comparison. The threshold was chosen based on the (“internal”) RMSE between the B3LYP-D3 geometries themselves, where the smallest difference between unique dimers was 1.20 Å. The 0.2 Å threshold was chosen to allow for accurate sorting taking into account small deviations in RMSE that may lead to incorrect assignment of dimers. Figure 5 shows the identified dimer from each optimization in B3LYP-D3, FFLUX, and IQA + LJ runs, as well as the RMSE of the FFLUX and IQA + LJ dimers compared to the B3LYP-D3-optimized geometry when there was a match. The RMSEs of the optimized geometries were approximately an order of magnitude smaller than the threshold.

FFLUX and IQA + LJ performed similarly yet again but with FFLUX having slightly a better accuracy in obtaining the correct optimized structure, obtaining the same as the B3LYP-D3 optimization 74% of the time compared to IQA + LJ’s 71%. This suggests that there could be a small benefit to using flexible multipole moments when simulating molecules that have a more rigid structure, like formamide, when starting away from minimum energy geometries.

#### 3.2.4 Bond Length Changes on Hydrogen Bond Formation

When forming hydrogen bonds, amides tend to show lengthening of the C=O bonds and shortening the C–N bonds. In a previous study of formamide, conducted at a lower level of theory than the current work, the C=O and C–N bond lengths were seen to increase by 0.010 and decrease by 0.015 Å, respectively,^52^ reflecting changes that had been seen experimentally on going from the gas^53^ to solid phase.^54^ If the hydrogen bonding were to be modeled well, these changes in bond length should also be captured in a calculation, a feature that should be possible in FFLUX but not in force fields like IQA + LJ where a rigid body constraint is imposed for static multipole moments.

To test whether the GPR model was able to predict these changes, differences in bond lengths between the monomer and dimers obtained from FFLUX optimizations were compared to the differences in the B3LYP-D3/aug-cc-pVTZ geometries. Table 3 shows that the changes in FFLUX geometries closely align with the changes seen in the B3LYP-D3/aug-cc-pVTZ geometries, with an average difference between the methods of 0.001 Å and a maximum difference of 0.004 Å for the C=O bonds in dimer *A*.

**Table 3:** Difference in the Lengths of the C=O and C–N Bonds Participating in Hydrogen Bonding in the Formamide Dimers Relative to the Monomer^a^

	C=O				C–N			
dimer	*B3LYP*	Δ	*FFLUX*	Δ	*B3LYP*	Δ	*FFLUX*	Δ
monomer	1.211		1.211		1.357		1.357	
*A*	1.227	0.016	1.223	0.012	1.338	–0.019	1.340	–0.017
*B*	1.224	0.013	1.223	0.012	1.345	–0.012	1.348	–0.009
*C*	1.219	0.008	1.219	0.008	1.358	0.001	1.358	0.001
*D*	1.218	0.007	1.218	0.007	1.350	–0.007	1.352	–0.005
*E*	1.218	0.007	1.217	0.006				

a All values are given in Å. In dimer *E*, the nitrogen atoms of the C–N bonds are not involved in hydrogen bonding.

Qualitatively, FFLUX predicts the changes in C=O and C–N bond lengths with high accuracy, the only difference being that the changes in the C=O bond lengths are predicted to be the same in dimers *A* and *B* while DFT predicts them to differ by 0.003 Å. Similarly, the relative changes in the C–N lengths are also captured in the correct order.

Interestingly, the C–N bond in dimer *C* shows an increase in length unlike the other dimers, which decrease in length upon formation of the C=O···HN hydrogen bond. A possible reason for this was thought to be the acceptance of a hydrogen bond at the nitrogen atom having a competing lengthening effect. However, this interaction also happens in dimer *D*, which shows a decrease in length. To better understand the effect of the acceptance of a hydrogen bond at the nitrogen atom in dimers *C* and *D*, the PES associated with the N···H separation in both dimers was investigated by performing relaxed scans in GAUSSIAN, perturbing the optimized separations by ±1Å in steps of 0.2 Å while keeping the C=O···HN hydrogen bond lengths constant.

**Figure 6:**
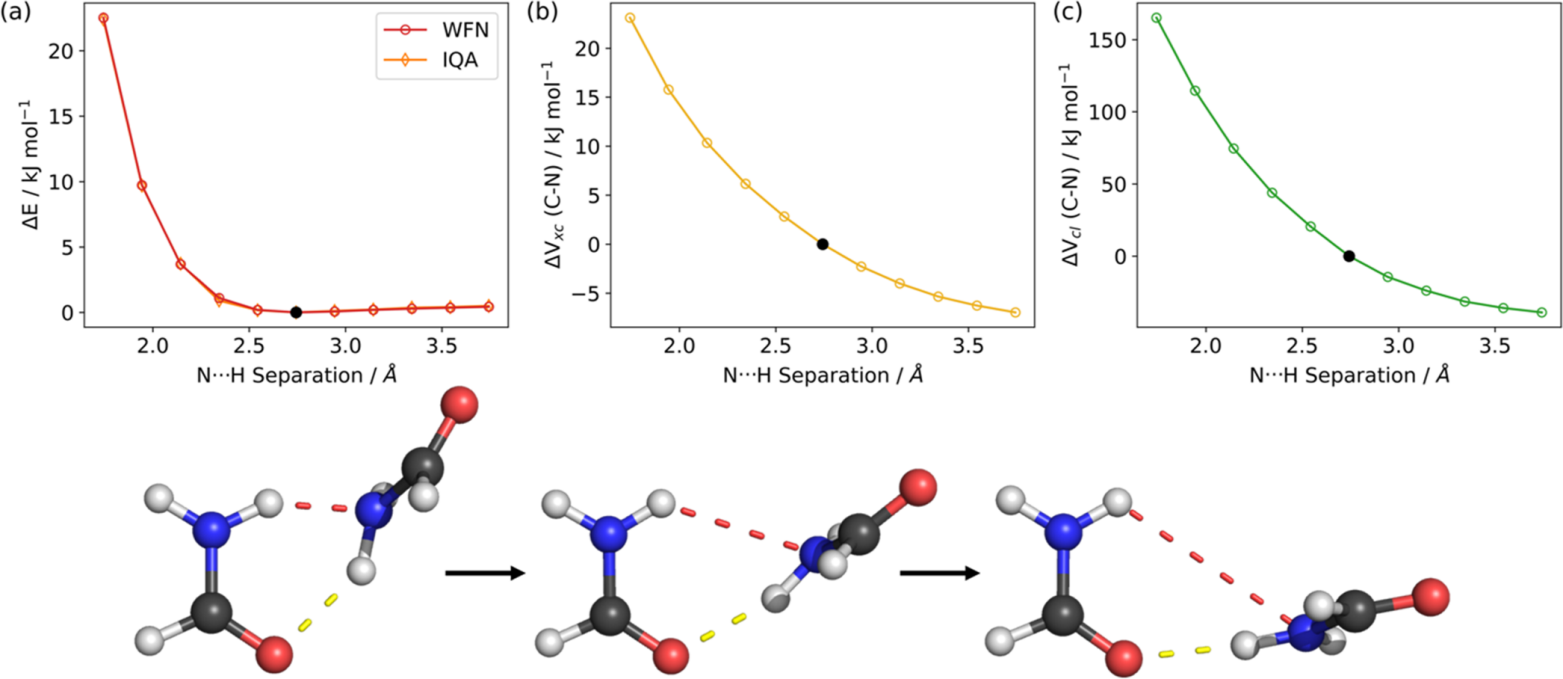
PES scan of the NH···N hydrogen-bond distance (red dash) in dimer *C* performed at the B3LYP/aug-cc-pVTZ level of theory. (a) Change in the wavefunction energy (red) and IQA total energy (orange) as a function of the N···H separation. (b) Change in the *V*_xc_ energy and (c) change in the *V*_cl_ energy associated with the C–N bond of the right-hand monomer over the scan coordinate. All energies are given relative to the energy in the optimized dimer, which is represented by the black dot in each panel.

**Figure 7:**
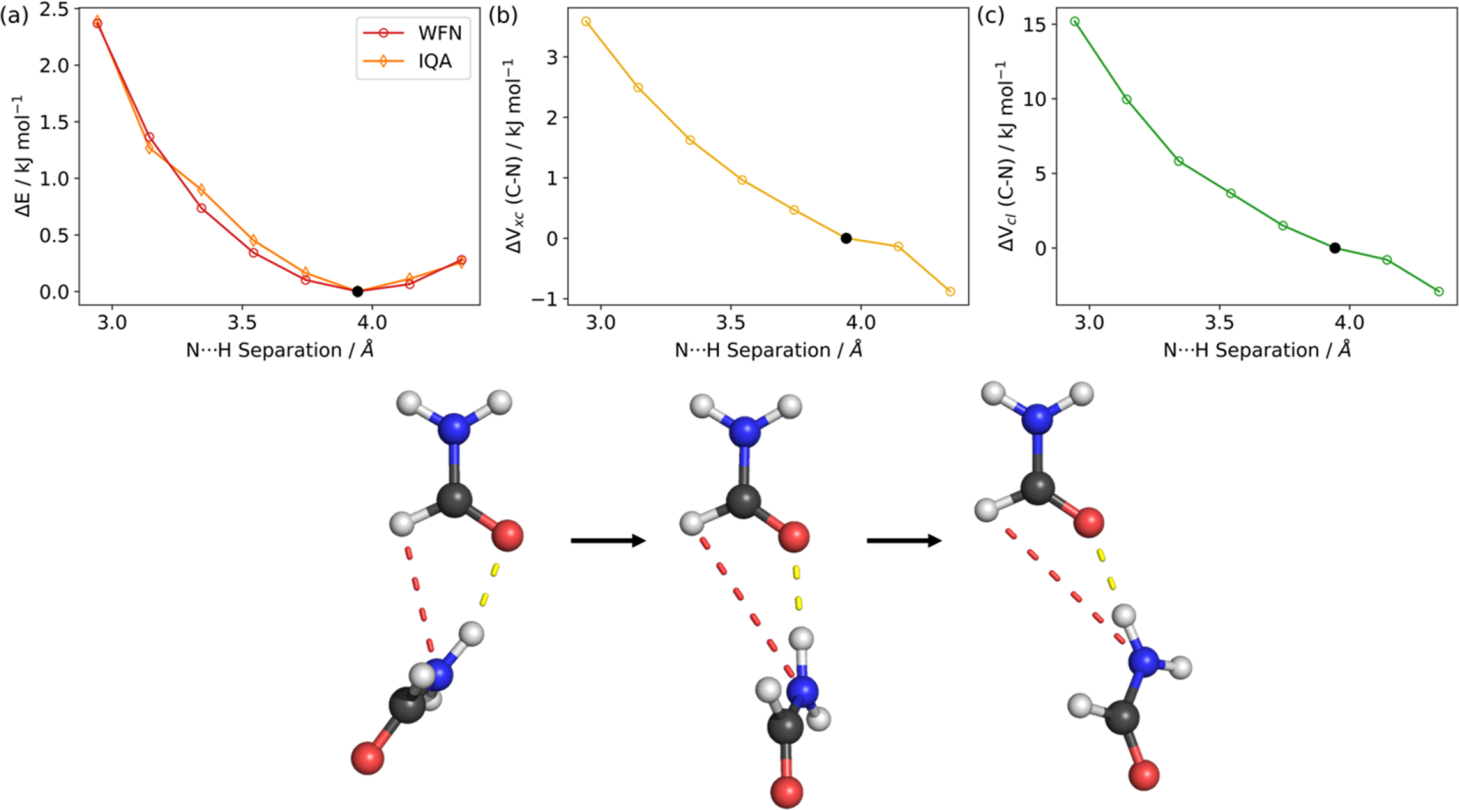
PES scan of the CH···N hydrogen-bond distance (red dash) in dimer *D* performed at the B3LYP/aug-cc-pVTZ level of theory. (a) Change in the wavefunction energy (red) and IQA total energy (orange) as a function of the N···H separation. (b) Change in the *V*_xc_ energy and (c) change in the *V*_cl_ energy associated with the C–N bond (bottom) over the scan coordinate. All energies are given relative to the energy in the optimized dimer, which is represented by the black dot in each panel. Note that this scan was truncated at larger separations due to significant structural changes during the relaxations.

The changes in the bond lengths can be understood through the changes in the *V*_xc_ and *V*_cl_ terms available from the IQA decomposition (see eq 5). *V*_xc_ describes the nonclassical contributions to the interaction between two atoms, i.e., the delocalization of electrons across a chemical bond, and can therefore be interpreted as a measure of the degree of covalency between a pair of atoms.^55−57^ On the other hand, *V*_cl_ describes the classical electrostatic interactions between two atoms. The scans performed for dimers *C* and *D* are shown in Figures 6 and 7, respectively, along with the interatomic *V*_xc_ and *V*_cl_ energies associated with the C–N bonds.

The PES obtained from each of the scans is shown in Figures 6a and 7a. Comparing the wavefunction energy with the IQA total energies shows excellent correspondence and a minimal mean absolute recovery error of less than 0.1 kJ mol^–1^ for both dimers. Panels (b) and (c), respectively, show the corresponding changes in the *V*_xc_ and *V*_cl_ associated with the C–N bond across the scans.

In both dimers *C* and *D*, there is an increase in the *V*_xc_ and *V*_cl_ energy terms between carbon and nitrogen, as the N···H separation decreases, that is, while the hydrogen bond is formed. This energy increase corresponds to a destabilization that would favor lengthening of the C–N bond. This confirms that the increase in C–N length seen in dimer *C* is caused by the acceptance of the hydrogen bond at the nitrogen atom, which counteracts the shortening from the formation of the C=O···H hydrogen bond. Dimer *D* may not show an increase despite having a similar interaction in its geometry due to it having a larger separation for the N···H interaction. Given that *V*_xc_ and *V*_cl_ are both proportional to 1/*r*_*ij*_ where *r*_*ij*_ is the separation between two atoms, the larger N···H separation in dimer *D* means that destabilization of the C–N bond due to the N···H interaction is less prominent than in *C*. This results in the shortening of the C–N bond in dimer *D*.

Ultimately, the changes in bond length seen in FFLUX are largely consistent with those seen in the B3LYP-D3/aug-cc-pVTZ DFT calculations, a feature that multipolar force fields that utilize rigid bodies would not be able to capture. This shows that the GPR model used for predicting the atomic energies and multipole moments in FFLUX is effectively “seeing” the electrons.

## 4 Conclusions

In this work the novel force field FFLUX, based on the principles of Quantum Chemical Topology, has been validated further on formamide. While previous work with FFLUX has mostly focused on water,^42,44,58^ here formamide was simulated for the first time with just a change of GPR model and nonbonded parameters and no major changes to the force field implementation.

The GPR model constructed with 1506 training points at the B3LYP/aug-cc-pVTZ level of theory had a maximum energy prediction error of 0.8 kJ mol^–1^ with 50% of predictions with errors less than 0.07 kJ mol^–1^, both well within the nominal threshold for chemical accuracy. The molecular dipole moment is predicted with errors below 0.1% relative to the training level of theory. Geometry optimization of the formamide monomer leads to a structure differing in energy from the DFT calculation by only 0.1 kJ mol^–1^ (only 2 × 10^–5^%). This shows the level of accuracy possible with FFLUX.

The monomeric model was found to be transferable to simulations on dimers with appropriate nonbonded parameters. When used in MD simulations with FFLUX, the five dimer structures reported in the literature were recovered with RMSEs of less than 0.1 Å relative to the *ab initio* optimized geometries. The performance was similar to that of a force field utilizing static multipole moments suggesting that for rigid molecules like formamide flexible moments may offer limited improvements. However, we expect this to not be the case for flexible molecules.

Changes in C=O and C–N bond lengths induced by the formation of hydrogen bonds, a subtlety not available to rigid body models, are also in close agreement with the B3LYP-D3/aug-cc-pVTZ DFT calculations. This result was obtained despite there being no explicit hydrogen bonding potential in the FFLUX simulations, which demonstrates that FFLUX effectively “sees” the electrons due to the GPR model.

The energy ranking predicted by FFLUX is consistent with B3LYP-D3/aug-cc-pVTZ with relative energies differing by less than 1 kcal mol^–1^ from the DFT calculations. Given the accuracy of the model in terms of its intramolecular energy and electrostatics, we suspect differences are due to our chosen Lennard-Jones parameters.

Looking at the future, models of clusters would in principle allow the GPR model to predict interactions between molecules, thereby negating the need for nonbonded potentials and allowing FFLUX to predict results even closer to the training level of theory. This would also allow us to capture many-body effects and long-range polarization. The simulation of dimers using the monomeric model is also a stepping stone to simulations on crystal structures, which will be presented in future work.

## Data Availability

The data that support the findings of this study are available from the corresponding author upon request.

## Supporting Information Available

Coordinates of the B3LYP/aug-cc-pVTZ optimized formamide monomer (Table S1.1) and the five dimers (Tables S1.2–S1.6) with corresponding ball and stick images (Figures S1.1–S1.6); S-curves for the formamide charge model (Figure S2.1); *S*-curves for the components of the formamide dipole moment (Figure S2.2); difference in bond lengths (Table S3.1) and angles (Tables S3.2) in the B3LYP/aug-cc-pVTZ and FFLUX-optimized monomers; IQA moments used in IQA + LJ optimizations; nonbonded parameters used in the FFLUX simulations (Table S5.1); and relative energies of the formamide dimers calculated with B3LYP/aug-cc-pVTZ and FFLUX (Table S6.1) (PDF)
