# Supplementary material for: Construction of a Gaussian Process Regression Model of Formamide for Use in Molecular Simulations
Source: J Phys Chem A. 2023 Feb 9;127(7):1702–14. doi: 10.1021/acs.jpca.2c06566 (PMC9969515; doi:10.1021/acs.jpca.2c06566)
Supplement: Supplementary file 1 — jp2c06566_si_001.pdf [file jp2c06566_si_001.pdf]

Supporting Information

Construction of a Gaussian Process Regression Model of Formamide for Use in Molecular Simulations

Matthew L. Brown, Jonathan M. Skelton and Paul L. A. Popelier*

Department of Chemistry, The University of Manchester, Oxford Road,

Manchester, M13 9PL, Britain

*Phone: +44 161 3064511. E-mail: pla@manchester.ac.uk

Contents

1. Coordinates of the B3LYP/aug-cc-pVTZ optimised geometries
2. Additional S-curves
3. Comparison of bond lengths and angles in the formamide monomer
4. IQA moments used in IQA+LJ optimisations
5. Non-bonded parameters used in the FFLUX calculations
6. Relative energies of the formamide dimers
7. References

1. Coordinates of the B3LYP/aug-cc-pVTZ optimised geometries

The formamide monomer and five dimers (A-E) were optimised in GAUSSIAN09[1] at the B3LYP/aug-cc-pVTZ level of theory. The coordinates of these optimised geometries are given in the Tables S1.1 to S1.5, each table being accompanied by the corresponding figure in the range Figures S1.1 to S1.5.

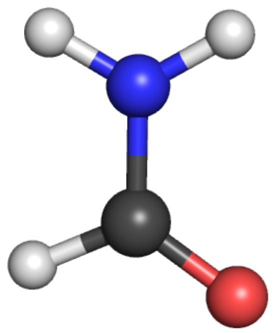

Figure S1.1. Structure of the formamide monomer.

Table S1.1. Coordinates of the formamide monomer optimised at the B3LYP/aug-cc-pVTZ level of theory.

C	0.7057	9.823661	4.962573
O	1.177176	8.711076	4.888886
N	0.732405	10.745375	3.966950
H	0.199029	10.197236	5.869591
H	1.169969	10.515932	3.089758
H	0.323432	11.654349	4.087233

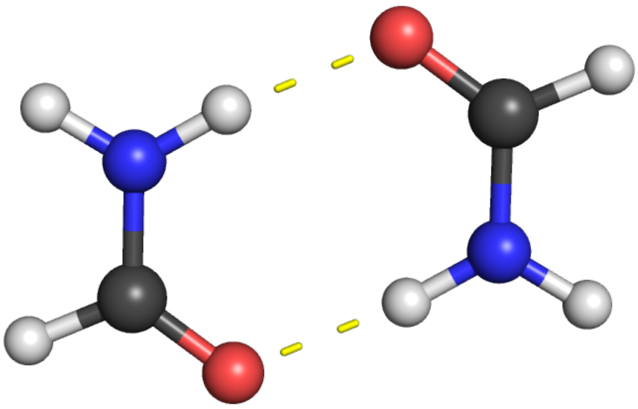

Figure S1.2. Structure of formamide dimer A.

Table S1.2. Coordinates of formamide dimer A optimised at the B3LYP-D3/aug-cc-pVTZ level of theory.

C	0.082014	-1.225621	-1.236365
O	-0.058474	-0.544468	-0.225931
N	0.215094	-0.746987	-2.478381
HC	0.107742	-2.324627	-1.175433
HN	0.206538	0.26431	-2.653021
HN	0.326128	-1.383757	-3.246734
C	0.024825	2.772294	-1.739567
O	0.16532	2.091137	-2.749996
N	-0.108186	2.293676	-0.497538
HC	-0.001007	3.871298	-1.800517
HN	-0.099548	1.28239	-0.322867
HN	-0.219266	2.930456	0.270801

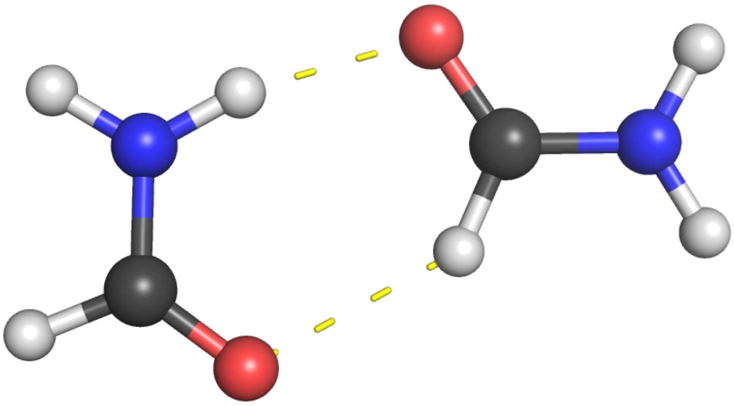

Figure S1.3. Structure of formamide dimer B.

Table S1.3. Coordinates of formamide dimer B optimised at the B3LYP-D3/aug-cc-pVTZ level of theory.

C	-4.707454	2.732117	-0.068126
O	-5.571134	1.869883	-0.0465
N	-3.382042	2.504536	-0.062765
HC	-4.964866	3.80464	-0.094675
HN	-3.020797	1.547736	-0.039091
HN	-2.743193	3.278769	-0.082184
C	-3.860673	-0.840471	0.020907
O	-2.761562	-0.303089	0.007476
N	-4.039163	-2.17608	0.055007
HC	-4.789871	-0.254036	0.005926
HN	-3.240869	-2.789331	0.070089
HN	-4.962085	-2.571005	0.063796

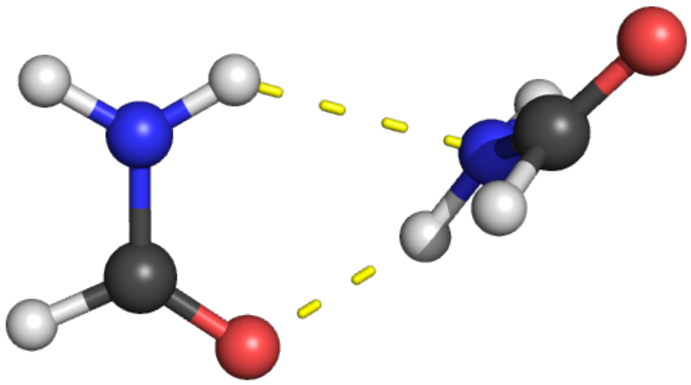

Figure S1.4. Structure of formamide dimer C.

Table S1.4. Coordinates of formamide dimer C optimised at the B3LYP-D3/aug-cc-pVTZ level of theory.

C	0.332515	-2.708691	1.398801
O	-0.555495	-3.317076	1.957995
N	0.278355	-2.229234	0.129825
HC	1.305575	-2.508764	1.880064
HN	-0.612923	-2.246717	-0.340238
HN	1.007337	-1.609607	-0.213066
C	3.399321	-2.025409	-1.353565
O	2.751355	-1.077698	-0.942902
N	2.976115	-3.305722	-1.359384
HC	4.412713	-1.900565	-1.764413
HN	2.060759	-3.532489	-0.999105
HN	3.565143	-4.041149	-1.705833

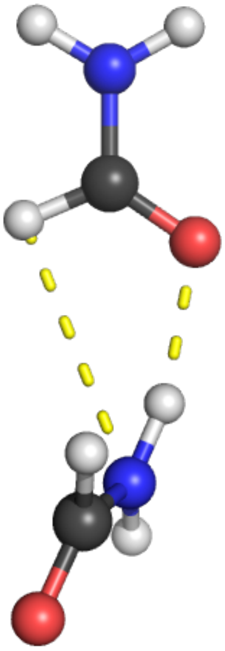

Figure S1.5. Structure of formamide dimer D.

Table S1.5. Coordinates of formamide dimer D optimised at the B3LYP-D3/aug-cc-pVTZ level of theory.

C	-1.797085	0.005752	-0.010434
O	-2.430363	-0.824037	-0.633105
N	-0.511719	-0.115674	0.382966
HC	-2.236991	0.971798	0.295429
HN	-0.023079	-0.971608	0.178899
HN	-0.053862	0.618743	0.912257
C	0.808931	3.133756	0.700722
O	0.685378	2.283386	1.5639
N	1.268568	4.381204	0.91947
HC	0.548277	2.944866	-0.352288
HN	1.531653	4.658532	1.851082
HN	1.345682	5.045373	0.170401

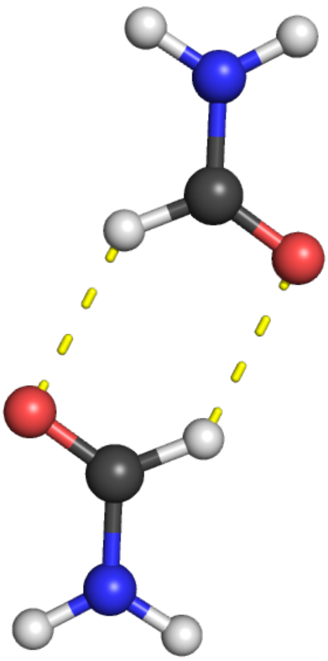

Figure S1.6. Structure of formamide dimer E.

Table S1.6. Coordinates of formamide dimer E optimised at the B3LYP-D3/aug-cc-pVTZ level of theory.

C	-6.301822	2.202545	-0.171578
O	-5.958059	3.106527	-0.911723
N	-7.585571	1.802134	-0.008773
HC	-5.581353	1.632212	0.432832
HN	-8.320916	2.263661	-0.518062
HN	-7.817147	1.051314	0.615919
C	-2.917654	2.499916	0.113898
O	-3.261411	1.594831	0.852723
N	-1.634164	2.901778	-0.047013
HC	-3.638128	3.070135	-0.490567
HN	-0.898873	2.439983	0.462111
HN	-1.402522	3.652924	-0.671288

2. Additional S-curves

Figure S2.1 shows the S-curves for the charges and Figure S2.2 those of the three components of the dipole moment. Multipole moments are learnt and predicted in the spherical tensor form; the components of the dipole moment shown in Figure S2.2 are therefore given in this form.

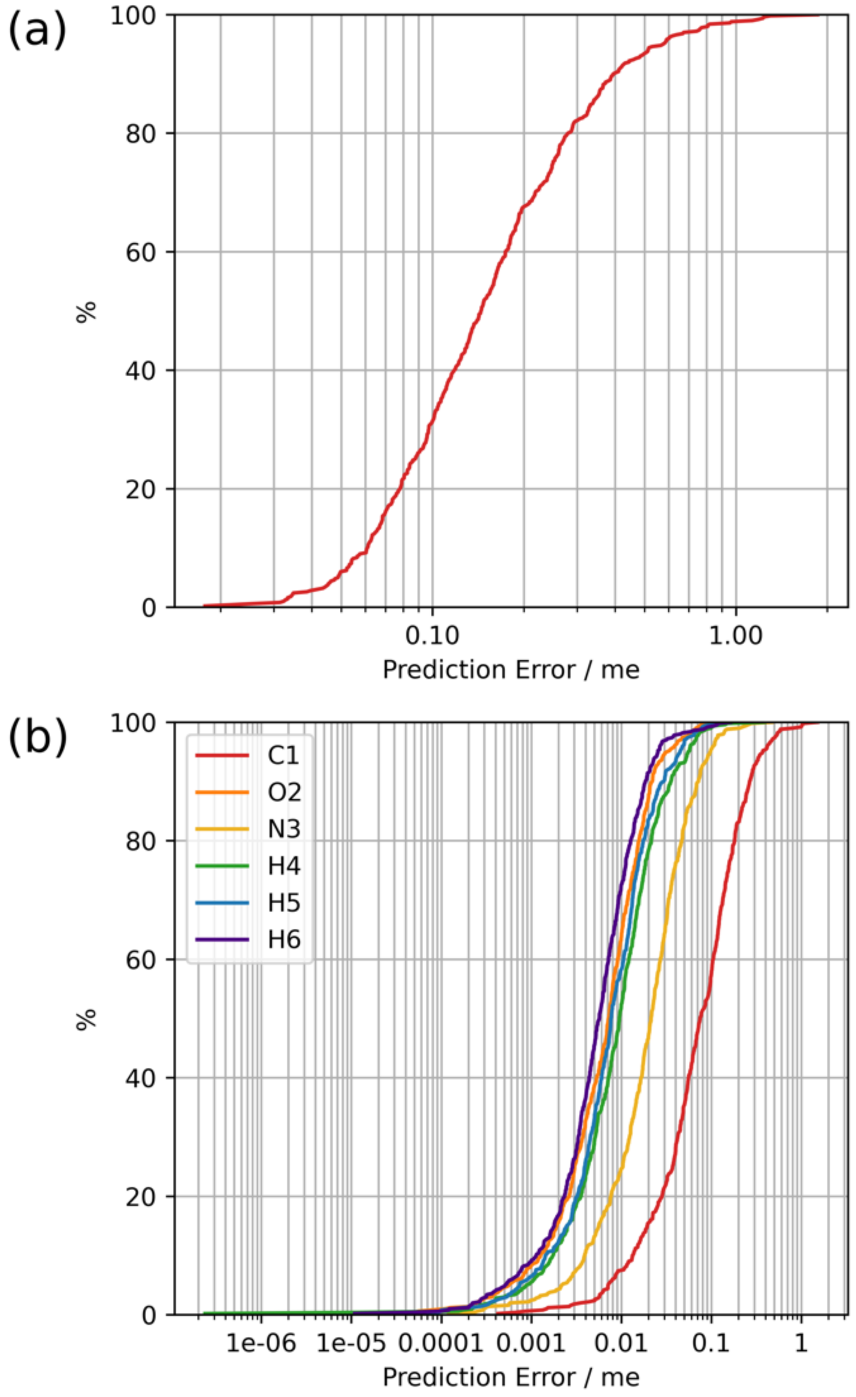

Figure S2.1. S-curves showing the absolute charge prediction errors (in milli-electron, me) for the 1,506-point formamide model for (a) the whole formamide molecule, and (b) for each constituent atom.

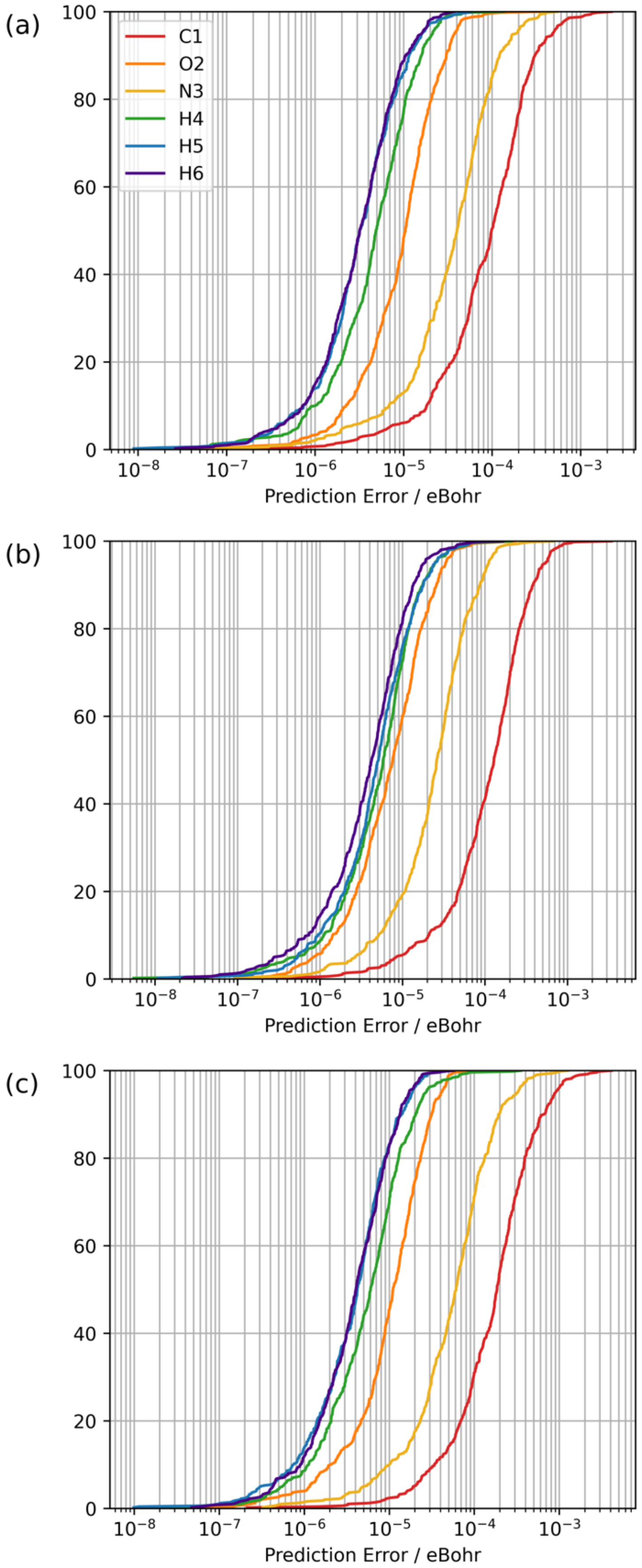

Figure S2.2. S-curves showing the absolute charge prediction errors for the 1,506-point formamide model for the (a) Q_{10} , (b) Q_{11c} , and (c) Q_{11s} component of the dipole moment (i.e. z, x and y).

3. Comparison of bond lengths and angles in the formamide monomer

Optimisation of the formamide monomer was performed to show the accuracy of the GPR model. The energy was found to differ from B3LYP/aug-cc-pVTZ by only 0.1 kJ mol^{-1}. The bond lengths (Table S3.1) and angles (Table S3.2) in the B3LYP and FFLUX optimised monomers are given, along with the differences between them. The maximum difference for bond lengths is 6.2×10^{-4} Å and 0.0567 degrees for angles suggesting that FFLUX predicts the potential energy surface of the monomer very well.

Table S3.1. Bond lengths in the B3LYP- and FFLUX-optimised monomers and the absolute difference between them. All values given in Å. Atom labels in parentheses indicate which atom the *H* is *cis* to.

Bond	B3LYP	FFLUX	Δ
C=O	1.21061	1.21068	0.00007
C-N	1.35703	1.35713	0.00010
C-H	1.10406	1.10396	0.00010
N-H(O)	1.00676	1.00646	0.00030
N-H(H)	1.00397	1.00335	0.00062

Table S3.1. Angles in the B3LYP- and FFLUX-optimised monomers and the absolute difference between them. All values given in degrees. Atom labels in parentheses indicate which atom the *H* is *cis* to.

Angle	B3LYP	FFLUX	Δ
C-N-H(O)	119.5387	119.5151	0.0236
C-N-H(H)	121.2671	121.2104	0.0567
O-C-H	122.6631	122.6945	0.0314
O-C-N	124.8826	124.8595	0.0231
N-C-H	112.4541	112.446	0.0081
H-N-H	119.1942	119.228	0.0338

4. IQA moments used in IQA+LJ optimisations

Multipole moments used in the IQA+LJ optimisations were calculated by partitioning the B3LYP/aug-cc-pVTZ optimised monomer using the interacting quantum atoms (IQA) partitioning scheme. Moments for each atom are given below in the form:

ATOM

Charge: q

Dipole: μ_x μ_y μ_z

Quadrupole: Θ_{xx} Θ_{xy} Θ_{xz} Θ_{yy} Θ_{yz} Θ_{zz}

Octupole: Ω_{xxx} Ω_{xxy} Ω_{xxz} Ω_{xyy} Ω_{xyz} Ω_{xzz} Ω_{yyy} Ω_{yyz} Ω_{yzz} Ω_{zzz}

where values are given in the default units for DL_POLY (protons Å^{-l} , where l corresponds to the rank of the multipole moment and 1 proton = $1.602*10^{-19}$ C).

C

1.527

-0.085 -0.394 0.000

-0.297 0.010 0.000 -0.465 0.000 -0.608

-0.020 -0.112 0.000 -0.014 0.000 -0.022 -0.145 0.000 -0.125 0.000

O

-1.178

-0.203 -0.118 0.000

-1.322 0.054 0.000 -1.398 0.000 -1.415

0.088 0.010 0.000 0.028 0.000 -0.027 0.070 0.000 -0.024 0.000

N

-1.199

0.060 -0.047 0.000

-1.235 0.025 0.000 -1.213 0.000 -1.608

-0.096 0.077 0.000 -0.008 0.000 -0.049 -0.066 0.000 -0.011 0.000

HC

0.022

-0.018 -0.081 0.000

-0.165 -0.005 0.000 -0.118 0.000 -0.184

-0.015 -0.040 0.000 -0.007 0.000 -0.007 -0.086 0.000 -0.044 0.000

HN

0.420

-0.012 0.091 0.000

-0.075 0.000 0.000 -0.066 0.000 -0.077

-0.012 0.025 0.000 0.000 0.000 -0.004 0.051 0.000 0.029 0.000

HN

0.408

-0.077 -0.053 -0.000

-0.071 0.005 0.000 -0.074 0.000 -0.079

-0.051 -0.004 0.000 -0.015 0.000 -0.025 -0.041 0.000 -0.017 0.000

5. Non-bonded parameters used in the FFLUX calculations

Parameter sets from the FIT[2-3], GAFF[4], Hagler[5-6], OPLS/AA[7-8] and W99[9] force fields were initially tested as non-bonded parameters for FFLUX simulations of dimers. All parameter sets used for the 12-6 potential are for the expression

$$U(r_{ij}) = \frac{A_{ij}}{r_{ij}^{12}} - \frac{B_{ij}}{r_{ij}^{6}} \tag{1}$$

except for FIT and W99, which used a Buckingham potential. Out of these sets, the Hagler parameters (*A* and *B*) performed the best, resulting in the fewest crashes where the parameters were too attractive and led to atoms falling into an infinitely attractive potential well. The Hagler set found four of the five minima (all but *E*) while OPLS/AA found *B, D*, and *E.* The other parameter sets were too attractive. While the Hagler parameter set succeeded in finding the most minima, it also generated more accurate geometries of *B* and *D* based on RMSEs than OPLS/AA did, making this the choice of parameter set to start from.

The original Hagler parameters were adapted for the L' = 3 simulations described in the main text. The FFLUX geometries obtained using the initial Hagler parameter set were inspected, looking at hydrogen bond lengths and angles to identify which atom-atom interactions could be too attractive or repulsive. The A (repulsion) and B (dispersion) values were then changed and a series of optimisations with different parameters were run. After optimisation RMSE values of the geometries were calculated relative to the B3LYP/aug-cc-pVTZ geometries, and the geometries from the parameter set that produced the lowest RMSE for the most dimers were inspected and the process was repeated. The final parameters are shown in Table S3.1.

Table S5.1. Non-bonded parameters used in FFLUX simulations.

Atom	A / Å^{12} kJ mol^{-1}	B / Å^{6} kJ mol^{-1}
C	13,534,048	12,606.560
O	3,440,600	3,900.368
N	10,891,864	11,486.320

For non-bonded interactions between different atoms the following mixing rules were used:

$$A_{ij} = \sqrt{A_i A_j} \tag{2}$$

$$B_{ij} = \sqrt{B_i B_j} \tag{3}$$

where i and j are atom types. These mixing rules were used in the Hagler paper[5] where the parameters were described initially. The values *A* and *B* are not the same as σ and ε in a typical Lennard-Jones potential but *A* and *B* can be calculated from them. The paper does mention that these combination rules and the Lorentz-Berthelot rules are not identical but find little difference between them.

6. Relative energies of the formamide dimers

Table S6.1. Relative energies of formamide dimers (A-E) calculated using B3LYP-D3/aug-cc-pVTZ and FFLUX.

**Dimer**	**B3LYP-D3/aug-cc-pVTZ (kJ mol^{-1})**	**FFLUX (kJ mol^{-1})**	**IQA+LJ (kJ mol^{-1})**
A	0.0	0.0	0.0
B	21.4	24.8	25.8
C	32.1	29.5	29.5
D	35.1	38.7	38.6
E	42.7	46.1	45.9

7. References

1. Frisch, M. J.; Trucks, G. W.; Schlegel, H. B.; Scuseria, G. E.; Robb, M. A.; Cheeseman, J. R.; Scalmani, G.; Barone, V.; Mennucci, B.; Petersson, G. A., GAUSSIAN09, revision B.01. *Gaussian Inc., Wallingford,* CT, USA **2010**.

2. Price, S. L.; Leslie, M.; Welch, G. W. A.; Habgood, M.; Price, L. S.; Karamertzanis, P. G.; Day, G. M., Modelling organic crystal structures using distributed multipole and polarizability-based model intermolecular potentials. *Phys.Chem.Chem.Phys.* **2010,** *12* (30), 8478-8490.

3. Beyer, T.; Price, S. L., Dimer or Catemer? Low-Energy Crystal Packings for Small Carboxylic Acids. *J.Phys.Chem.B.* **2000,** *104*, 2647-2655.

4. Wang, J., Wolf, Romain M., Caldwell, James W., Kollman, Peter A., Case, David A., Development and testing of a general amber force field. *J.Comp.Chem.* **2004,** *25* (9), 1157-1174.

5. Hagler, A.; Huler, E.; Lifson, S., Energy functions for peptides and proteins. I. Derivation of a consistent force field including the hydrogen bond from amide crystals. *J.Am.Chem.Soc.* **1974,** *96* (17), 5319-5327.

6. Hagler, A. T.; Lifson, S., Energy Functions for Peptides and Proteins. II. The Amide Hydrogen Bond and Calculation of Amide Crystal Properties. *J.Am.Chem.Soc.* **1974,** *96*, 5327–5335.

7. Jorgensen, W. L.; Maxwell, D. S.; Tirado-Rives, J., Development and testing of the OPLS all-atom force field on conformational energetics and properties of organic liquids. *J.Am.Chem.Soc.* **1996,** *118* (45), 11225-11236.

8. de la Luz, A. P.; Méndez-Maldonado, G. A.; Núñez-Rojas, E.; Bresme, F.; Alejandre, J., A New Force Field of Formamide and the Effect of the Dielectric Constant on Miscibility. *J.Chem.Theor.Comp.* **2015,** *11* (6), 2792-2800.

9. Williams, D. E., Improved intermolecular force field for molecules containing H, C, N, and O atoms, with application to nucleoside and peptide crystals. *J. Comp. Chem.* **2001,** *22* (11), 1154-1166.
